# Glutamine metabolism and the unfolded protein response in MYC-driven breast cancer

**DOI:** 10.1186/2049-3002-2-S1-P66

**Published:** 2014-05-28

**Authors:** Ayesha Shajahan-Haq, Katherine Cook, Jessica Schwartz-Roberts, Ahreej Eltayeb, Diane Demas, Anni Warri, Leena Hilakivi-Clarke, Robert Clarke

**Affiliations:** 1 Department of Oncology, Lombardi Comprehensive Cancer Center, Georgetown University Medical Center, Georgetown, Washington, DC, USA

## 

Antiestrogens are used to treat estrogen receptor positive (ER+) breast tumors that constitute 70% of all breast cancer cases. Unfortunately, acquired resistance to antiestrogen therapy remains a critical clinical obstacle. Here we show that human breast cancer cells and rat mammary tumors that have acquired resistance to antiestrogens express increased levels of MYC, a major regulator of both glutamine and glucose. Glutamine metabolism and glucose uptake were elevated in ER+ antiestrogen resistant cells (LCC9) compared with sensitive cells (LCC1). Inhibition of MYC, with siRNA or small molecule inhibitor, reduced cell viability and uptake of both glutamine and glucose in resistant cells. In resistant cells, MYC expression controlled protein levels of glutamine, glutamate and glucose transporters as well as GLUL and GLS, two enzymes that promote glutamate-glutamine inter-conversion. Increased MYC function in resistant cells correlated with increased cellular sensitivity to deprivation of, and also inhibitors of, both glutamine and glucose. While apoptosis eliminated all resistant cells in glucose-only conditions beyond 72 h, in glutamine-only conditions, the unfolded protein response (UPR) via GRP78-IRE1α and activating JNK and increased CHOP, induced apoptosis in majority of the cells but promoted survival in some. The antiestrogen faslodex (FAS; ICI 182,780) significantly reduced glucose uptake in antiestrogen resistant cells compared with sensitive cells. Thus, our findings reveal unique roles for MYC in promoting metabolic flexibility in and promoting survival in antiestrogen resistant breast cancer cells via the UPR. Targeting glutamine and glucose metabolism pathways, therefore, may provide novel strategies in treating endocrine resistant breast cancers.

